# Detection of Interactions between Proteins through Rotation Forest and Local Phase Quantization Descriptors

**DOI:** 10.3390/ijms17010021

**Published:** 2015-12-24

**Authors:** Leon Wong, Zhu-Hong You, Zhong Ming, Jianqiang Li, Xing Chen, Yu-An Huang

**Affiliations:** 1 College of Computer Science and Software Engineering, Shenzhen University, Shenzhen 518060, China; lg_wong@foxmail.com (L.W.); mingz@szu.edu.cn (Z.M.); lijq@szu.edu.cn (J.L.); yahuang1991@gmail.com (Y.-A.H.); 2 School of Computer Science and Technology, China University of Mining and Technology, Xuzhou 221116, China; 3 Academy of Mathematics and Systems Science, Chinese Academy of Sciences, Beijing 100190, China; xingchen@amss.ac.cn

**Keywords:** protein-protein interaction, Rotation Forest, Physicochemical Property Response Matrix (PR), Local Phase Quantization

## Abstract

Protein-Protein Interactions (PPIs) play a vital role in most cellular processes. Although many efforts have been devoted to detecting protein interactions by high-throughput experiments, these methods are obviously expensive and tedious. Targeting these inevitable disadvantages, this study develops a novel computational method to predict PPIs using information on protein sequences, which is highly efficient and accurate. The improvement mainly comes from the use of the Rotation Forest (RF) classifier and the Local Phase Quantization (LPQ) descriptor from the Physicochemical Property Response (PR) Matrix of protein amino acids. When performed on three PPI datasets including *Saccharomyces cerevisiae*, *Homo sapiens*, and *Helicobacter pylori*, we obtained good results of average accuracies of 93.8%, 97.96%, and 89.47%, which are much better than in previous studies. Extensive validations have also been explored to evaluate the performance of the Rotation Forest ensemble classifier with the state-of-the-art Support Vector Machine classifier. These promising results indicate that the proposed method might play a complementary role for future proteomics research.

## 1. Introduction

As a necessary component of all organisms, proteins are involved in most processes of living cells. Because proteins usually function in pairs, knowledge of protein interactions can provide great insights into more biological functions [1,2]. As a hotspot in proteomics research, detecting protein-protein interactions (PPIs) is conducive to understanding disease mechanisms and making progress in developing drugs for specific diseases. In recent years, many innovative techniques based on biological experiments have been developed for detecting PPIs. The valuable PPI data on diverse species have been accumulated by high-throughput experimental technologies, such as protein chip [3,4], yeast two-hybrid (Y2H) [5,6,7] systems, tandem affinity purification (TAP) [8], mass spectrometry protein complex identification (MS-PCI) [9] and correlated mRNA expression profiling [10]. Further studies are boosted by this available data even though the current PPI data obtained through biological approaches cover only a small fraction of the complete PPI network [11,12]. When adopting a given experimental method exposes its inevitable disadvantages of high costs in time, money, and labor, but poor performance with high rates of false negatives and false positives [13–16]. Thus, computational approaches for predicting protein-protein interactions are good complementary techniques to experimental methods [17].

Many computational methods have been developed for predicting PPIs. They are based on different data sources, such as gene fusion, sequence conservation among interacting proteins, gene neighborhood, literature mining knowledge, phylogenetic profiles, and combining interaction information from various data sources [18]. However, these methods suffer from the need for previous knowledge of proteins and their performance is sensitive to the reliability of the previous information. In addition, with the development of genomic technologies, the amount of protein sequence data from various species has been growing exponentially. Therefore, researchers have recently proposed some computational methods for predicting PPIs based on the knowledge of protein amino acids sequences without the inclusion of any additional information. These computational validations indicate the feasibility of predicting PPIs using protein amino acid sequences alone [19,20,21].

Among these previous works, Zhou *et al.* [22] proposed a computational method based on the support vector machine (SVM) and uses auto-correlation descriptors and correlation coefficients. Gough and Bock [23] proposed to combine SVM with structural and physiochemical descriptors. Shen *et al.* [20] used SVM as a classifier and applied the conjoint triad method for feature extraction, in which the 20 amino acids are divided into seven categories according to the volumes and dipoles of their side chains. Thanathamathee and Lursinsap [24] employed the *proteinproplot* algorithm for feature extraction from protein sequences. They reduced the dimensions of features using principle component analysis (PCA) and adopted the feed-forward neural network as a classifier. Guo *et al.* [12] applied the auto-covariance method to detect the correlation among segments from the non-continuous amino acids sequence. Although these approaches show some promise, there is still room for improvement with regard to efficiency and accuracy.

In this paper, we present a sequence-based computational method for detecting PPIs that combines the Rotation Forest classifier with a novel matrix-based protein sequence representation. More specifically, the Physicochemical Property Response Matrix (PR) method is applied to represent the amino acid sequence as a matrix corresponding to a physicochemical property. In addition, the Local Phase Quantization (LPQ) method is used to extract the features from the PR matrix that contain useful coefficients. In the final step, we apply the Rotation Forest prediction model to predict PPIs. To verify the effectiveness and feasibility, we evaluate the proposed method on three prevalent datasets, including *Saccharomyces cerevisiae*, *Homo sapiens*, and *Helicobacter pylori*. The experimental analysis shows that the proposed method can extract more key information beyond the protein sequence itself and can approach higher prediction accuracy compared with previous methods. The rest of this paper is organized as follows. In Section 2, we present the evaluation measures and the parameter selection criteria. In addition, a comparison with other methods is presented to demonstrate the advantages of the proposed method. Section 3 shows the datasets and methods used for PPI prediction. Finally, we draw some conclusions in Section 4.

## 2. Results and Discussion

### 2.1. Evaluation Measures

For the purpose of measuring the prediction performance of the proposed method, overall Accuracy, Sensitivity, Precision, Matthews Correlation Coefficient (MCC), and Receiver Operating Characteristic (ROC) and Area Under Curve (AUC) were calculated. The definitions of these measures are as follows:

(1) $$Accuracy=\frac{TP+TN}{TP+FP+TN+FN}\;$$

(2) $$Sensitivity=\frac{TP}{TP+FN}\;$$

(3) $$Precision=\frac{TP}{TP+FP}$$

(4) $$MCC=\frac{\text{TP}\times \text{TN}-\text{FP}\times \text{FN}}{\sqrt{\left(TP+FN\right)\times \left(TN+FP\right)\times \left(TP+FP\right)\times \left(TN+FN\right)}}$$

where true positive (TP) is where the testing samples, having PPIs, are predicted successfully; false negative (FN) is where the testing samples, non-interacting protein pairs, are predicted unsuccessfully; false positive (FP) is where the testing samples, having PPIs, are predicted unsuccessfully; true negative (TN) is where the testing samples, non-interacting protein pairs, are predicted successfully; Mathews correlation coefficient is the abbreviation of MCC that is a correlation coefficient that measures the quality of binary classifications in machine learning. In addition, ROC curve is a graphical plot with specificity-sensitivity for a binary classifier system. And AUC, a threshold independent measure, is to assess the performance by the normalized area under the ROC curve.

### 2.2. Parameter Selection

The number of feature subsets *K* and decision tree number *L* are crucial for the performance of the Rotation Forest classifier. Therefore, we need to set these two vital parameters in advance. It is quite complex to set the specific value and obtain the best performance for randomness and uncertainty. A higher value of *K* indicates more subsets, where each subset has fewer features, and a higher value of *L* indicates more basic classifiers in the ensemble classifier.

In this context, overall classification accuracy is evaluated on a *Helicobacter pylori* dataset using different *K* and *L* values in the first computational validation. Specifically, we adopt the parameter selection strategy that the first step is to fix *L* to 20 and tune *K* from 10 to 70 at intervals of 5. We then set *K* to the value obtained from the first step and tune *L* from 10 to 70 at intervals of 5.

**Figure 1:**
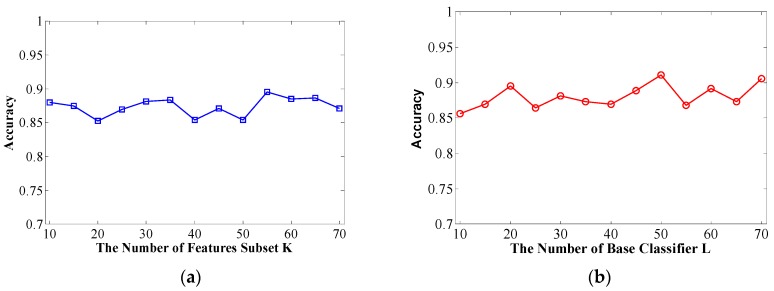
(**a**) Overall prediction accuracy rate with increasing *K* of feature subsets; (**b**) Overall prediction accuracy rate with increasing *L* of decision trees.

The prediction results of *Helicobacter pylori* are shown in Figure 1. From Figure 1a, we can see that setting the *K* = 55 leads can obtain good result with an accuracy of 89.54% on the conditions of *L* = 20. We then set *K* to 55 and increase the value of *L* from 10 to 70 at intervals of 5 to work out the results shown in Figure 1b. We then determine that the optimal value of *L* is 50.

The same parameter selection strategy is adopted when exploring the other two datasets. The proposed method on the Human dataset yields an accuracy of 97.91% with the optimized settings (*K* = 25; *L* = 40). For the *Saccharomyces cerevisiae* dataset, it achieves the best accuracy of 94.32% with the optimized settings (*K* = 65, *L* = 40).

### 2.3. Prediction Performance of Proposed Model

To validate the proposed model, we apply it to three prevalent PPIs datasets, including the *Helicobacter pylori* dataset, *Homo sapiens* dataset, and *Saccharomyces cerevisiae* dataset. To avoid the problem of over-fitting, five-fold cross-validation is used for performance evaluation. We also operate the support vector machine (SVM) to compare its performance with the proposed model.

**Figure 2:**
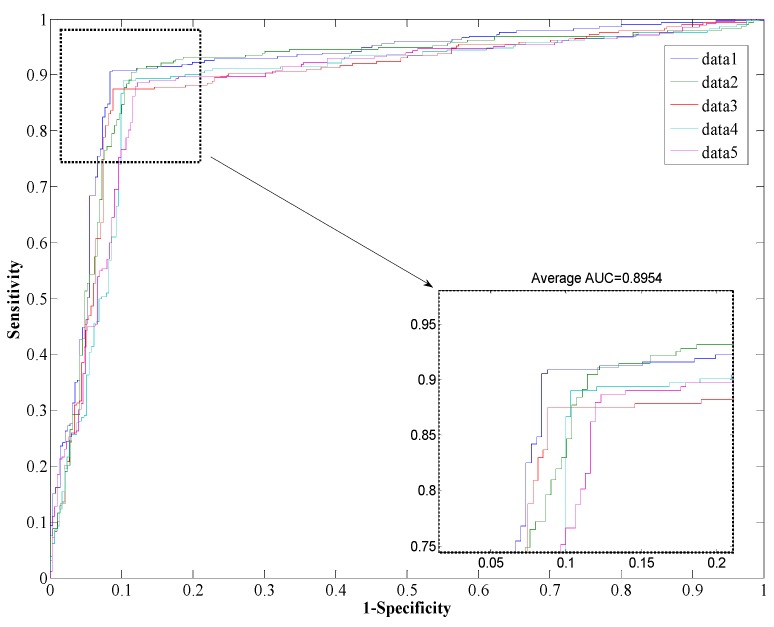
Receiver Operating Characteristic (ROC) from proposed method result for *H. pylori* protein-protein interaction (PPI) dataset.

**Figure 3:**
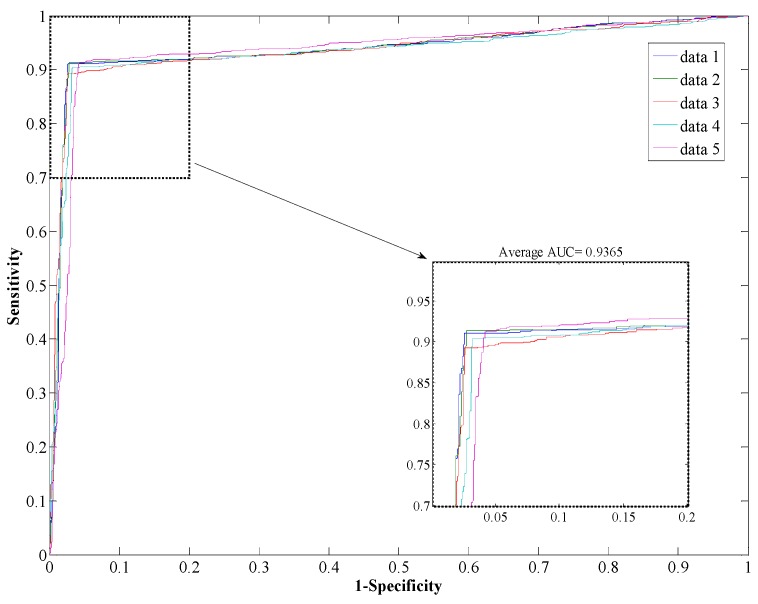
ROC from proposed method result for *S. cerevisiae* PPI dataset.

The performance of the *Helicobacter pylori* and *Saccharomyces cerevisiae* dataset are shown in Table 1 and Table 2, which list the overall accuracy, sensitivity, precision, MCC, and AUC. And the ROC curves are plotted in Figure 2 and Figure 3. We can see from Table 1 that the proposed method yields a high accuracy of 89.47% on average on the *Helicobacter pylori* dataset. The average value of the AUC is close to 0.90, which indicates the method has high precision in predicting PPIs. The standard deviation of the accuracy, precision, sensitivity, MCC, and AUC are 1.05%, 1.77%, 1.41%, 0.0167, and 0.0145, respectively. When employed on the *Saccharomyces cerevisiae* dataset, our proposed method yields an AUC of 0.93 with a high accuracy of 93.80%, and the values of precision and sensitivity are 96.66% and 90.64%, respectively. The standard deviations of accuracy, precision, sensitivity, MCC, and AUC are 0.50%, 0.62%, 0.87%, 0.009, and 0.002, respectively.

**Table 1:** The prediction results of the *H. pylori* dataset using the proposed method.

Test Set	Sensitivity (%)	Precision (%)	Accuracy (%)	MCC	AUC
1	90.57	91.81	91.08	0.8375	0.9158
2	90.48	88.96	89.54	0.8126	0.9048
3	87.15	90.61	89.19	0.8070	0.8896
4	89.04	89.66	89.37	0.8099	0.8823
5	88.65	87.11	88.16	0.7912	0.8842
Average	89.18 ± 1.42	89.63 ± 1.77	89.47 ± 1.05	0.81 ± 0.0167	0.90 ± 0.0145

**Table 2:** The prediction results of the *S. cerevisiae* dataset using the proposed method.

Test Set	Sensitivity (%)	Precision (%)	Accuracy (%)	MCC	AUC
1	89.22	97.16	93.34	0.8752	0.9381
2	91.18	95.61	93.47	0.8779	0.9387
3	90.40	96.61	93.52	0.8786	0.9368
4	91.07	97.06	94.32	0.8924	0.9331
5	91.34	96.88	94.37	0.8933	0.9358
Average	90.64 ± 0.87	96.66 ± 0.62	93.80 ± 0.50	0.88 ± 0.009	0.94 ± 0.002

**Figure 4:**
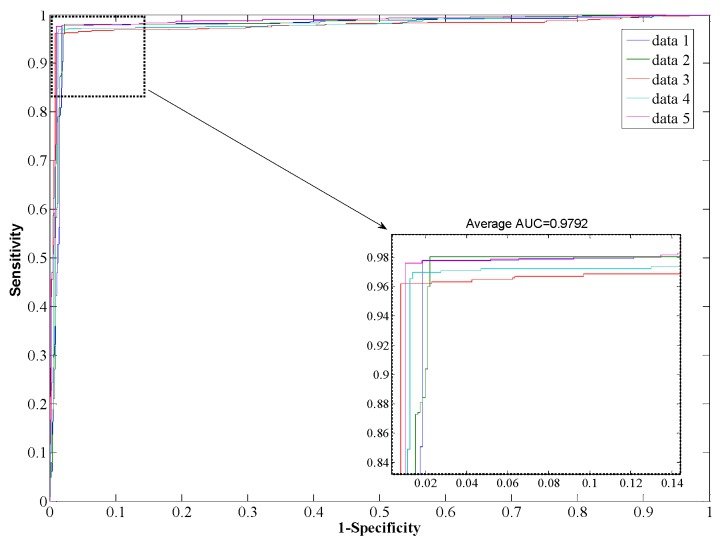
ROC from proposed method result for *Human* PPI dataset.

**Figure 5:**
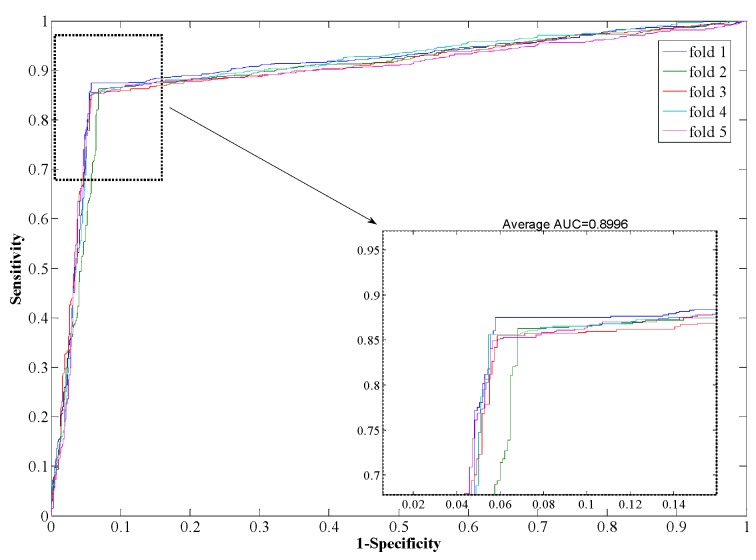
ROC from SVM-based method result for *Human* PPI dataset.

The Support Vector Machine (SVM) is a state-of-the-art classification model. Therefore, we compare the Rotation Forest classifier with the SVM model on the *Human* dataset. The experimental results are shown in Table 3, from which it can be seen that our proposed method yields good results reflected in average values of accuracy, precision, sensitivity, and MCC as high as 97.96%, 98.35%, 97.32%, and 0.96, respectively. When employing the SVM model for prediction, the average values of accuracy, precision, sensitivity, and MCC are 90.21%, 93.00%, 85.96%, and 0.82, respectively. From the ROC curves of Figure 4 and Figure 5, it can also be seen that the average AUC score of the proposed method was 0.9792, and the value of SVM was 0.8996. In addition, the standard deviations of accuracy, sensitivity, and MCC yielded by the proposed method were as low as 0.22%, 0.73%, and 0.0042, respectively, which are lower than the values obtained by the SVM model of 0.46%, 0.99%, and 0.0077, respectively. In conclusion, the experimental results above suggested that our proposed method is much better than the SVM-based method.

**Table 3:** The prediction results of the *Human* dataset using the proposed method compared with SVM.

Model	Test Set	Sensitivity (%)	Precision (%)	Accuracy (%)	MCC	AUC
Rotation Forest	1	97.68	97.93	97.91	0.9590	0.97.68
	2	98.07	97.57	97.91	0.9591	0.97.93
	3	96.21	99.06	97.79	0.9566	0.97.65
	4	96.98	98.40	97.85	0.9578	0.97.79
	5	97.64	98.80	98.34	0.9673	0.98.53
	Average	97.32 ± 0.73	98.35 ± 0.61	97.96 ± 0.22	0.96 ± 0.004	0.98 ± 0.004
SVM	1	87.52	93.59	90.92	0.8343	0.9055
	2	86.28	92.07	89.88	0.8170	0.8959
	3	85.46	93.00	90.01	0.8185	0.8985
	4	85.62	93.05	90.44	0.8244	0.9047
	5	84.93	93.27	89.82	0.8156	0.8935
	Average	85.96 ± 0.99	93.00 ± 0.57	90.21 ± 0.46	0.82 ± 0.008	0.90 ± 0.005

### 2.4. Comparison with Other Methods

Many methods have been proposed for predicting PPIs. Here, we compare the prediction performance of the proposed method with the existing approaches. All the results yielded by different methods on the *Saccharomyces cerevisiae* dataset are shown in Table 4. We can observe from Table 4 that Zhou’s work performs well with the lowest standard deviation of 0.33% for accuracy, and Guo’s work has a higher accuracy of 89.33%. In addition, Yang’s work makes a higher precision value of 90.24%. It should be noticed that the proposed method yields the best performance in terms of sensitivity, precision, accuracy and MCC at 90.64%, 96.66%, 93.80%, and 88.35%, respectively. The corresponding standard deviations are 0.87%, 0.62%, 0.50%, and 0.87%, respectively. The above results show that the performance of our proposed method is superior.

We also compare our proposed method with other methods on the *Helicobacter pylori* dataset and the results are shown in Table 5. Compared with the other methods, the proposed method achieves outstanding performance for its high sensitivity, precision, accuracy, and MCC. In detail, the performances of the classifiers are quite disparate. The worst result, yielded by the phylogenetic bootstrap, has an accuracy of 75.80%, precision of 80.20%, and sensitivity of 69.80%. HKNN achieves 84.00% accuracy, 84% precision, and 86% sensitivity. In contrast, the proposed method achieves an accuracy of 89.47%, precision of 89.63%, sensitivity of 89.18%, and an MCC of 81.16%, respectively. The above results indicate that our proposed method is promising and exhibits good performance for PPIs prediction.

**Table 4:** Comparison of other methods on the *S. cerevisiae* dataset.

Model	Test Set	Sensitivity (%)	Precision (%)	Accuracy (%)	MCC (%)
Zhou’s work	SVM + LD	87.37 ± 0.22	89.50 ± 0.60	88.56 ± 0.33	77.15 ± 0.68
Guo’s work	ACC	89.93 ± 3.68	88.87 ± 6.16	89.33 ± 2.67	*N/A*
	AC	87.30 ± 0.22	87.82 ± 4.33	87.36 ± 1.38	*N/A*
Yang’s work	Cod1	75.81 ± 1.20	74.75 ± 1.23	75.08 ± 1.13	*N/A*
	Cod2	76.77 ± 0.69	82.17 ± 1.35	80.04 ± 1.06	*N/A*
	Cod3	78.14 ± 0.90	81.86 ± 0.99	80.41 ± 0.47	*N/A*
	Cod4	81.03 ± 1.74	90.24 ± 1.34	86.15 ± 1.17	*N/A*
Proposed Method	Average	90.64 ± 0.87	96.66 ± 0.62	93.80 ± 0.50	88.35 ± 0.87

*N/A* means none available.

**Table 5:** Comparison of other methods on the *H. pylori* dataset.

Model	Sensitivity (%)	Precision (%)	Accuracy (%)	MCC (%)
Phylogenetic bootstrap	69.80	80.20	75.80	*N/A*
Boosting	80.37	81.69	79.52	70.64
Signature products	79.90	85.70	83.40	*N/A*
HKNN	86.00	84.00	84.00	*N/A*
Proposed Method	89.18	89.63	89.47	81.16

*N/A* means none available.

## 3. Materials and Methods

### 3.1. Generation of the Data Sets

The first dataset is derived from *Saccharomyces cerevisiae* in which we selected the core subset of the Database of Interacting Proteins (DIP). We implement a data preprocessing program to remove the redundant protein pairs. More specifically, protein pairs with more than forty percent sequence identity or fewer than fifty residues are removed. The final positive pairs are comprised of 5594 protein pairs, and the final negative pairs with different sub-cellular localizations have the same number as the positive pairs. The final dataset consists of 11,188 protein pairs.

The *Homo sapiens* dataset is generated from the Human Protein References Database (HPRD). The original dataset has 3899 interacting pairs and 4262 non-interacting pairs after filtering the ones with more than 25% sequence identity. More specifically, the interacting protein pairs are generated from 2502 different kinds of protein derived from humans. The non-interacting protein pairs are yielded from 661 kinds of proteins. However seven of the sequences are too long and exceed our computational ability when using the proposed protein presentation method. The final *Homo sapiens* dataset contains 3892 positive samples and 4262 negative samples. The *Helicobacter pylori* dataset is described by Martin *et al.* [25], which consists of 2916 protein pairs, of which half are positive and the rest negative.

### 3.2. Representation for Protein

To borrow the feature extraction techniques from image processing, it is necessary to preprocess each amino acid sequence by transforming them into a matrix. The method, named Physicochemical Property Response Matrix (PR) [21], is used to represent the protein sequence. First, the physicochemical property response matrix *PRM^d^*(*i*,*j*) ∈ *R_N×N_* is calculated for a given protein *P =* (*p_1_*, *p_2_*,…, *p_n_*) and its size depends on the protein sequence by selecting a specific physicochemical property *d*. According to a specific physicochemical property *d*, set the value in *PRM^d^*(*i*,*j*) to the sum of the indexing values corresponding to the amino acid in position *i* and *j*. Consider

(5) $$PRM\left(i,j\right)=index\left(p_{i}\right)+index\left(p_{j}\right)i,j=1,\ldots ,N$$

where *index*(*p*) denotes the value of the certain property in AAIndex for the protein amino acid *p*.

In the proposed method, we employ the hydrophobicity index as the physicochemical property. Table 6 shows the values of the hydrophobicity index for each amino acid. For instance, assuming the protein amino acids sequence *p* = “ARND”, then its PRM is as follows:

(6) $$PRM=\left[\begin{array}{cccc} \begin{array}{l} 0.61+0.61\\ 0.60+0.61\\ 0.06+0.61\\ 0.46+0.61 \end{array} & \begin{array}{l} 0.61+0.60\\ 0.60+0.60\\ 0.06+0.60\\ 0.46+0.60 \end{array} & \begin{array}{l} 0.61+0.06\\ 0.60+0.06\\ 0.06+0.06\\ 0.46+0.06 \end{array} & \begin{array}{l} 0.61+0.46\\ 0.60+0.46\\ 0.06+0.46\\ 0.46+0.46 \end{array} \end{array}\right]$$

After calculating the matrix, the matrix would be compressed if its size is larger than 250 × 250. Because the physicochemical property response matrix is two-dimensional and the amino acid sequences may be beyond the ability of our computer performance, a handful of sequences with excessive length would be ignored.

**Table 6:** The values of the hydrophobicity property for each amino acid.

Amino Acids	A	R	N	D	C	Q	E	G	H	I
Values	0.61	0.60	0.06	0.46	1.07	0	0.47	0.07	0.61	2.22
**Amino Acids**	**L**	**K**	**M**	**F**	**P**	**S**	**T**	**W**	**Y**	**V**
Values	1.53	1.15	1.18	2.02	1.95	0.05	0.05	2.65	1.88	1.32

### 3.3. Feature Vector Extraction

There are many methods of extracting features from images in image processing. The Local Phase Quantization (LPQ) [21] method is a common and efficient texture descriptor that adopts the *Fourier* transform to analyze the information in matrix. It is based on the blur invariance property of the Fourier phase spectrum. That is, the observed image is generated from the original image after blur processing. Consider

(7) g(x)=f(x)×h(x)

where *g*(*x*), *h*(*x*), and *f*(*x*) denote the observed image, original image and blur function, respectively. The Fourier transform functions of Function (4) are as follows:

(8) G(x)=F(x)×H(x)

where *G*(*x*), *H*(*x*), and *F*(*x*) are the Fourier transform functions of *g*(*x*), *h*(*x*), and *f*(*x*), respectively.

In the LPQ method, to reflect the local information effectively, the Fourier transform operates on the locality of the image that is on the neighborhood $N_{m\times m}$ located at *x* with the size of m×m. Consider

(9) $$F\left(u,x\right)=\underset{y\in N_{m\times m}}{\sum }f\left(x-y\right)e^{-j^{2}\pi u^{T}y}=w_{u}^{T}f_{x}$$

The local phase information is extracted from the two-dimensional short-term Fourier Transform (STFT). The STFT is used to calculate a rectangular neighborhood transformed from each pixel position. Because the output of Fourier transform and its phase are continuous, the LPQ method employs four kinds of phase. That is, it would output four complex coefficients that correspond to four field two-dimensional frequencies after STFT, and that contain a real part and imaginary part and then use a binary coding scheme to quantize them as integers between 0 and 255. Consider

(10) $$F_{x}^{c}=[F(u_{1},x),F(u_{2},x),F(u_{3},x),F(u_{4},x)].$$

and

(11) $$F_{x}={\left[Re\{F_{x}^{c}\},Im\{F_{x}^{c}\}\right]}^{T}$$

where *Re* and *Im* denote the real part and the imaginary part. The corresponding binary sequence is as follows:

(12) $$w={\left[Re\left\{w_{u1},w_{u2},w_{u3},w_{u4}\right\},Im\left\{w_{u1},w_{u2},w_{u3},w_{u4}\right\}\right]}^{T}.$$

The feature vector utilized in the experiment is a normalized histogram of such coefficients calculated from STFT. As a protein pair contains two parts, the final feature vector of an interaction pair is constructed by concatenating the descriptors of two proteins.

### 3.4. Rotation Forest

An ensemble classifier usually has higher performance than a single base classifier. In this study, a new classification model, Rotation Forest (RF), is employed to predict PPIs based on a novel quantitative description of the protein amino acid sequence. Rotation Forest exhibits excellent classification performance and is widely applied as classifier in data mining [26].

Assume a matrix *X*, size of *N × n*, denoted as *N* training samples, where each sample has *n* features. Set a label vector *Y =* [*y_1_*,*…*, *y_N_*]^T^ with size *N × 1* that holds the value 1 or −1 to differentiate whether the protein pair is interacting or not. A value of 1 represents PPI and −1 non-PPI. Denote K as the number of subsets of feature set F and L as the number of the decision trees in a Rotation Forest. An individual decision tree is denoted as *D_i_*. Note that the parameters *K* and *L* must be set in advance.

The training procedure for an individual decision tree classifier *D_i_* is as follows:

Step 1: Select *K* subsets from the feature set *F* at random. Note that each subset must hold *M* = *n/K* features, and fill with zero vectors if the last subset has less than *M* features.

Step 2: Set the *jth* feature subset to *F_ij_* for training classifier *D_i_*, and let *X_ij_* be a dataset of features in *F_ij_*. Then, denote a new set $X_{ij}^{\prime }$ in which three-fifths of the new training set describes a bootstrap subset of targets. Generate coefficients by applying PCA on $X_{ij}^{\prime }$, and store in matrix *C_ij_*, which is composed of the coefficients of principal components, $a_{ij}^{\left(1\right)}$,…, $a_{ij}^{M_{j}}$, where the size of each is *M* *× 1*.

Step 3: Organize *Cij*, and generate a sparse rotation matrix *Ri*, as follows:

(13) $$R_{i}=\left[\begin{array}{cccc} a_{i1}^{\left(1\right)},\ldots ,a_{i1}^{\left(M_{1}\right)} & \left\{0\right\} & \cdots & \left\{0\right\}\\ \left\{0\right\} & a_{i2}^{\left(1\right)},\ldots ,a_{i2}^{\left(M_{2}\right)} & \left\{0\right\} & \cdots \\ \vdots & \left\{0\right\} & \cdots & \cdots \\ \vdots & \cdots & \ddots & \cdots \\ \left\{0\right\} & \left\{0\right\} & \cdots & a_{iK}^{\left(1\right)},\ldots ,a_{iK}^{\left(M_{K}\right)} \end{array}\right]$$

It is essential to rearrange *R_i_* and build $R_{i}^{a}\left(sizeN\times n\right)$ to match the order of the feature set *F*. Finally, $\left(Y,XR_{i}^{a}\right)$ is allocated as the training set to train decision tree *D_i_*.

After training *L* decision trees, when a given test sample *x* is as input, each decision tree *D_i_* assigns the probability $d_{i,j}\left(xR_{i}^{a}\right)$ and assumes that sample *x* has correlation. Then, an average probability $\mu_{j}\left(x\right)$ is calculated as follows:

(14) $$\mu_{j}\left(x\right)=\frac{1}{L}\overset{L}{\underset{i=1}{\sum }}d_{i,j}\left(xR_{i}^{a}\right)\;j=1,\ldots ,c.$$

Finally, assign *x* to the class with the highest confidence.

## 4. Conclusions

Predicting interactions between protein pairs is of great importance to understand the molecular basis of complex cellular processes. This article reports a novel computational method for predicting protein-protein interactions solely using the protein sequence. Three large, real public PPI data sets including *Saccharomyces cerevisiae*, *Homo sapiens*, and *Helicobacter pylori* datasets are explored to evaluate the prediction performance of the proposed method. The validation results show that our proposed model can achieve better performance than the existing methods. The improvement of our method mainly comes from the use of the Rotation Forest (RF) classifier and the Local Phase Quantization (LPQ) descriptor from the Physicochemical Property Response Matrix (PR). Therefore, the proposed method can be used to guide related experimental validations and as a supplementary tool to proteomics research.
